# Is consolidative thoracic radiotherapy of extensive-stage small cell lung cancer still beneficial in the era of immunotherapy? A retrospective analysis

**DOI:** 10.1007/s00066-023-02075-9

**Published:** 2023-04-11

**Authors:** Elgin Hoffmann, Chiara De-Colle, Vlatko Potkrajcic, David Baumann, Werner Spengler, Cihan Gani, David Utz

**Affiliations:** 1 Department of Radiation Oncology, Eberhard-Karls University Tübingen, 72076 Tübingen, Germany; 2 Department of Medical Oncology and Pneumology, Eberhard-Karls University Tübingen, 72076 Tübingen, Germany

**Keywords:** Small cell lung cancer, extensive stage, Chemoimmunotherapy, Consolidative thoracic radiotherapy, Radiotherapy and immunotherapy, PD-L1 inhibitor

## Abstract

**Purpose:**

Extensive-stage small cell lung cancer (ES-SCLC) carries a dismal prognosis. The benefit of consolidative thoracic radiotherapy (TR) after first-line chemoimmunotherapy with PD-L1 inhibitors in this setting remains unclear. As TR can improve overall survival (OS) after conventional chemotherapy, we retrospectively analyzed OS of an inhouse cohort treated either with TR or with chemoimmunotherapy alone.

**Methods:**

A total of 41 patients treated with chemoimmunotherapy with PD-L1 inhibitors (atezolizumab or durvalumab) for ES-SCLC at our hospital since 2019 were analyzed. TR was administered in 10 fractions of 3 Gy. Patient characteristics, number of immunotherapy cycles received, brain irradiation, and presence of hepatic and cerebral metastasis at diagnosis were assessed. Primary endpoint was OS after first diagnosis.

**Results:**

Consolidative TR was associated with a significantly longer OS than systemic therapy alone (1-year OS 78.6% and 2‑year OS 37.1% vs. 1‑year OS 39.7% and 2 years not reached, *p* = 0.019). With regard to radiotherapy indication, survival at 1 year was 88.9% (log-rank *p* = 0.016) for patients receiving consolidative TR. For patients receiving TR in case of progression, 1‑year survival was 66.7%. Hepatic and cerebral metastasis at first diagnosis had no significant effect on OS.

**Conclusion:**

TR was significantly associated with longer OS. The survival benefit of TR was most pronounced for consolidative radiotherapy after initial chemoimmunotherapy compared to TR in case of progression. Although retrospective findings need to be interpreted with caution, in the absence of prospective data, our findings provide a basis for offering consolidative TR in the era of chemoimmunotherapy.

## Background

Small cell lung cancer (SCLC) constitutes about 20–25% of all lung tumors and carries a dismal prognosis, especially in case of extensive stage (ES) [1, 2]. Two thirds of patients present with ES at first diagnosis, associated with a 2-year survival rate of 5–10%. Research efforts have thus been aimed at improving the poor prognosis of ES-SCLC.

In case of good response after palliative polychemotherapy in this situation, consolidative thoracic radiotherapy (TR) can improve overall survival in selected patient groups [3, 4]. Recommended dose and fractionation range from 30 Gy in 10 fractions up to definitive doses in patients with very good prognostic factors [5].

More recently, the addition of programmed death-ligand (PD-L1) inhibitors such as atezolizumab and durvalumab to established chemotherapy has led to an improvement in overall survival in patients with ES-SCLC, as demonstrated in the IMpower-133 trial and the CASPIAN trial [6–8]. Current European Society for Medical Oncology (ESMO) guidelines state platin-based polychemotherapy with etoposide in combination with a PD-L1-inhibitor followed by maintenance immunotherapy as first-line treatment for patients with good performance status and no contraindication against immunotherapy [9]. However, consolidative thoracic/mediastinal radiotherapy is not routinely recommended after chemoimmunotherapy as it was not included in either of the approval studies (IMpower-133 and CASPIAN).

So far, the effect of consolidative TR in addition to chemoimmunotherapy has only been studied retrospectively in a small cohort comparing 20 patients who received thoracic/mediastinal radiotherapy after atezolizumab in addition to platin-based chemotherapy to a historic control [10]. Toxicity analysis in this study found no increase in radiation-related toxicity, especially no increase in pneumonitis, and overall survival was comparable to historic controls. Two prospective studies comparing the effect of additional TR in ES-SCLC patients—the RAPTOR-trial (NCT04402788) and the TREASURE trial [11]—have been proposed, but are currently still recruiting.

However, a direct comparison between patients receiving consolidative TR after combined chemoimmunotherapy and patients treated with chemoimmunotherapy alone has not yet been published. In this situation, the benefit of consolidative TR remains unclear. Our retrospective analysis was therefore aimed at assessing the overall survival of patients with ES-SCLC who received TR either in case of stable disease or local response to systemic therapy, or in case of progression after initial good response, compared to patients receiving systemic chemoimmunotherapy only. The presence of hepatic and cerebral metastasis at initial diagnosis, the application of whole-brain irradiation, and patient characteristics were also analyzed.

## Methods

### Patient selection

All patients treated with first-line combined chemoimmunotherapy for extensive-stage small cell lung cancer at our university hospital between June 2019 up until the start of analyses in November 2022 were screened. As neuroendocrine tumors of the lung also comprise large cell neuroendocrine carcinoma and dedifferentiated atypical carcinoid tumors, and the clinical course of disease in these entities mirrors that of SCLC and patients received the same treatment regimen, all patients with advanced neuroendocrine tumors of the lung were eligible. Only patients with extensive disease at first diagnosis who received at least one dose of immunotherapy in addition to platin-based palliative chemotherapy during the first three systemic therapy cycles, after which a CT-based staging assessing treatment response was conducted, were included in the analysis. Consolidative TR was considered in patients with good or partial response of the primary as well as stable disease. In case of local progression, the indication for radiotherapy was discussed on a case-by-case basis. Broad consent to analysis of clinical data and outcome had been given by all patients included in the analysis. The study was approved by the ethics committee of the University of Tuebingen (project no. 751/2022BO2), and analyses were conducted in compliance with the declaration of Helsinki.

### Treatment

Indication for TR as consolidation after first-line systemic treatment, in case of progression, or no indication for radiotherapy, as well as time interval between first diagnosis and start of mediastinal radiotherapy, were assessed. The application of brain irradiation (prophylactic cranial irradiation [PCI] or whole-brain radiotherapy [WB-RT]) was also analyzed. The following patient characteristics were determined: age at diagnosis, gender, number of immunotherapy cycles received, presence of hepatic and cerebral metastasis at diagnosis, neuron specific enolase (NSE) at diagnosis, radiotherapy regimen, other radiotherapy treatment, and treatment regimen in case of progression. ECOG was not routinely reported. Primary endpoint was overall survival after initial diagnosis.

All patients were discussed in the interdisciplinary tumor board. Due to lack of clear data regarding the benefit of consolidative TR, the decision for mediastinal irradiation was based on discussion in the interdisciplinary tumor board and patients’ decision. Generally, radiotherapy was offered patients either in case of a response of the primary tumor and responsive or stable metastatic burden (if present) after combined chemoimmunotherapy after the first re-staging or in case of progression of the primary tumor and stable metastatic burden under maintenance immunotherapy. Metastases per se were not a contraindication for radiotherapy. PCI or therapeutic whole-brain irradiation prior or parallel to TR was permitted. Thoracic radiotherapy planning was CT based and treatment was delivered as IMRT. Patients were treated with 30 Gy in 10 fractions according to the CREST trial fractionation [3]. Radiotherapy planning was conducted accordingly and comprised the post-chemotherapy volume of the primary tumor site as well as hilar and mediastinal lymph nodes that were affected prior to chemotherapy. Planning target volume (PTV) margin was set at 15 mm. According to our institutional standards, mean lung dose was not to exceed 20 Gy and volume receiving 20 Gy (V20 Gy) had to be < 35%. Assessment of lung function was mandatory before start of radiotherapy and estimated post-radiotherapy forced expiratory volume in one second (FEV1) needed to be at least 1 l. Cerebral radiotherapy was delivered as 3D conformal radiotherapy to the whole brain in 10 fractions of 2.5 to 3.0 Gy. In few selected cases, it was delivered as stereotactic radiotherapy to the resection cavity (five fractions of 6 Gy) and/or single metastases (single dose 18–20 Gy).

### Statistical analysis

Statistical analyses were conducted using SPSS (IBM Corp. Released 2021. IBM SPSS Statistics for Windows, version 28.0. IBM Corp, Armonk, NY, USA). For descriptive statistics, chi-squared and Fisher’s exact tests were employed. Group comparison was conducted using *t*-tests with differences between groups of *p* < 0.05 considered significant. Overall survival was calculated according to the Kaplan–Meier method using log-rank tests and was defined as the time from the date of first diagnosis until death of any cause. Due to the comparatively small size of the cohort, a matched pair analysis could not be performed. To assess the impact of possible confounding factors on overall survival, Cox regression analyses were conducted for cerebral radiotherapy as well as the presence of hepatic and cerebral metastasis at first diagnosis. Significance threshold was set at *p* = 0.05.

## Results

### Patient characteristics

In total, 55 patients were eligible for analysis. Two patients were not included as they were receiving radiotherapy parallel to the analysis, so that 53 patients were analyzed. However, 12 patients died, dropped out before completion of three cycles of systemic therapy and at least one cycle of immunotherapy, or showed progression before the first re-staging, so that 41 patients were eligible for survival analysis. Patient characteristics of the whole cohort are detailed in Table 1. One patient with large cell neuroendocrine carcinoma and one patient presenting with an aggressive and rapidly progressing atypical carcinoid tumor of the lung were also included. 23 patients received mediastinal radiotherapy. Characteristics of groups receiving consolidating TR vs. no TR are listed in Table 2. Median follow-up was 12.5 months (SD = 7.3 months) owing to an average survival over all groups of 12.7 months (SD = 7.4 months).

**Table 1:** Patients’ characteristics

Male vs. female				22 male	19 female
Age at diagnosis (years)				62.43	SD = 8.39
Histology SCLC				39	
Other neuroendocrine tumor of the lung				2	
Atezolizumab				32	
Durvalumab				9	
Number of cycles				8.2	SD 6.41
Number of cycles (all patients with at least two cycles)				8.9	SD 6.28
Discontinuation because of side effects				5	
Only one cycle of immunotherapy				4	
Mediastinal radiotherapy				23	
After good response/stable disease				14	
After local progression				9

**Table 2:** Group comparison between patients receiving thoracic radiotherapy and non-irradiated patients

		Thoracic radiotherapy		No thoracic radiotherapy		
		23		18		
Male vs. female		11 vs. 12	47.8%	11 vs. 7	61.1%	*p* = 0.298
Age at diagnosis (years)		62.61	8.99	62.20	7.80	*p* = 0.880
NSE at diagnosis (μg/l)		100.29	SD 169.61	174.00	SD 335.46	*p* = 0.236
Atezolizumab vs. durvalumab		21 vs. 2	91.3%	11 vs. 7	57.9%	*p* = 0.026*
Less than two cycles of immunotherapy		2	8.7%	2	11.1%	*p* = 0.598
Adverse effects of immunotherapy		4	17.4%	1	5.6%	*p* = 0.258
Immunotherapy cycles received		9.5	SD 7.5	6.5	SD 4.6	*p* = 0.060
Hepatic metastasis		10	43.5%	9	47.4%	*p* = 0.460
Cerebral metastasis		7	30.4%	10	52.6%	*p* = 0.097
Both cerebral and hepatic metastasis		4	17.4%	3	16.7%	*p* = 0.642
Cerebral irradiation		17	73.9%	9	47.4%	*p* = 0.106
Time until start of thoracic radiotherapy (months)		7.44	SD 3.96	–	–	–
Time until start of cerebral radiotherapy (months)		6.48	SD 2.88	4.32	SD 3.72	*p* = 0.084

*NSE* neuron-specific enolase, *SD* standard deviation

Descriptive statistics of categorical variables performed using single-sided Fisher’s exact test, for all other comparisons, single-sided student’s *t*-test was used. Statistical results were considered significant for *p* < 0.05. Statistically significant differences between groups are marked with*

### Chemoimmunotherapy

All patients received platin-based chemotherapy with either carboplatin or cisplatin together with etoposide. At least one cycle of atezolizumab or durvalumab was administered, in most cases from the second or third cycle onwards. Immunotherapy with PD-L1 inhibitors was discontinued in case of adverse effects or disease progression. Adverse effects of immunotherapy occurred in seven cases (17.1% of all patients) and included allergic reaction, diabetes, polyneuropathy, pneumonitis, and adrenal insufficiency. Minor toxicity to radiotherapy was not well documented and could not be assessed. No discontinuation of immunotherapy because of radiation-induced pneumonitis was reported. For disease progression, a second-line chemotherapy with topotecan was initiated or patients were re-exposed to a platin-based chemotherapy. On average, patients received 8.2 cycles of combined chemoimmunotherapy, including maintenance therapy (range 1 to 31 cycles, SD 6.4 cycles). 37 patients (90.2%) received two or more cycles of PD-L1 inhibitors.

### Dose and fractionation

In most patients (86.9%), radiotherapy was conducted with 30 Gy in 10 fractions. Three patients (13.0%) received an individual concept with stereotactic radiotherapy of residual disease with single doses of 6 to 15 Gy in 5 or 3 fractions, in case of residual pleural disease manifestation without lymph node involvement of the mediastinum or very limited mediastinal lymph node affection, respectively. In 28 patients, cerebral radiotherapy was administered, 11 cases of which (39.2%) received PCI. 6 patients who were treated with TR also received WB-RT because of cerebral metastasis (26.1%). 13 patients (46.4% of all patients) received only cerebral radiotherapy, whereas 6 patients (26.1%) treated with TR did not receive additional cerebral radiotherapy.

### Survival analysis

**Fig. 1:**
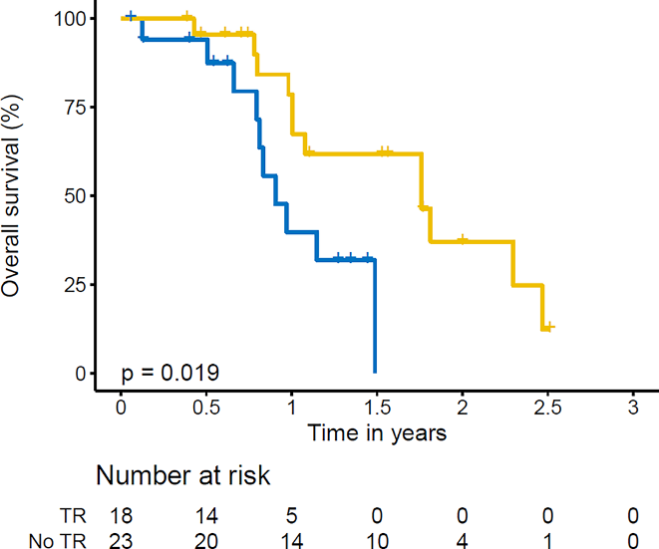
Overall survival with regard to thoracic radiotherapy (TR). Overall survival of patients who received no thoracic radiotherapy (*blue line*, no TR) versus overall survival of patients who received thoracic radiotherapy (*yellow line*, TR) regardless of radiotherapy indication and who completed at least one cycle of chemoimmunotherapy. Statistical threshold was set at *p* = 0.05

**Fig. 2:**
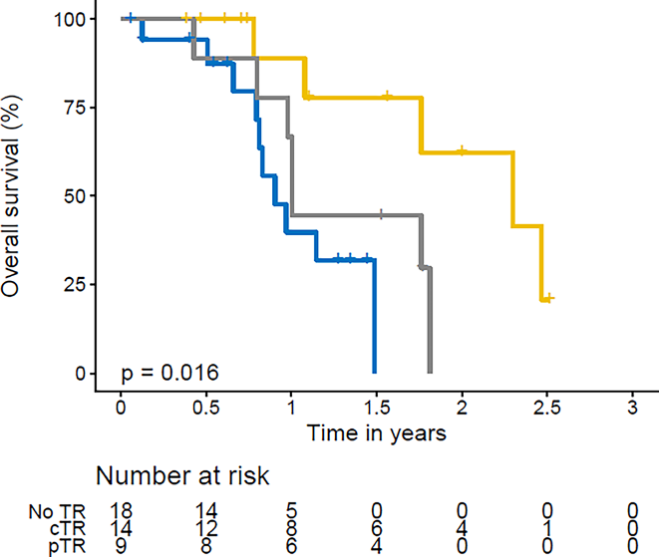
Overall survival with regard to thoracic radiotherapy (*TR*) indication. Patients who did not receive TR (*blue line*, no TR) versus overall survival of those receiving TR in case of progression (*grey line*, pTR) and those receiving TR for consolidation (*yellow line*, cTR). Statistical threshold was set at *p* = 0.05

Kaplan–Meier curves detailing overall survival curves for patients receiving TR vs. patients who received chemoimmunotherapy only are depicted in Fig. 1. Overall survival was significantly longer in the group of patients treated with TR: For the whole cohort, 1‑year OS after TR was 78.6% vs. 39.7%. Median survival was 21.1 months and 10.8 months, respectively (95% CI 12.24–30.0 vs. 8.04–13.68, log rank *p* = 0.019). Kaplan–Meier curves detailing overall survival with regard to indication for TR—i.e., consolidation vs. in case of progression—or no radiotherapy are shown in Fig. 2. There was a significant difference in overall survival for patients receiving consolidative TR: survival at one year was 88.9% (log-rank *p* = 0.016) with a median survival of 27.6 months (95% CI 15.0–40.2 months). For patients receiving TR in case of progression, 1‑year survival was at 66.7% with a median survival of 12.0 months (95% CI 11.64–12.48 months).

### Cox regression analysis

Cox regression analysis was conducted to assess the influence of cerebral radiotherapy, NSE level, cerebral metastasis, and hepatic metastasis at first diagnosis on overall survival. Thoracic radiotherapy and brain irradiation were associated with a longer overall survival, but this association was not significant (thoracic radiotherapy: HR 0.39, 95% CI 0.14–1.052, *p* = 0.063; WB-RT HR 0.43, 95% CI 0.17–1.08, *p* = 0.073). In case of the presence of cerebral metastasis at first diagnosis, there was no significant association with overall survival (cerebral metastasis: HR 2.02, 95% CI 0.76–5.4, *p* = 0.156; TR: HR 0.38, 95% CI 0.14–1.06, *p* = 0.064). Likewise, the presence of hepatic metastasis at first diagnosis did not have a significant impact on overall survival, with an HR 2.40 (95% CI 0.94–6.16, *p* = 0.069) with a sustained association for longer overall survival after TR (HR 0.33, 95% CI 0.12–0.88, *p* = 0.026).

## Discussion

In ES-SCLC, TR after platin-based chemotherapy can yield a survival benefit in certain patient groups [3, 4, 12]. In the era of immunotherapy, the benefit of TR in this setting is unclear, as it was not part of the IMpower-133 and CASPIAN approval studies for PD-L1-inhibitors [6–8]. Therefore, it is not routinely recommended for ES-SCLC in NCCN, ESMO, and national guidelines [5, 9]. In detail, German national guidelines do not comment on TR in ES-SCLC, neither as consolidation after chemoimmunotherapy or in case of progression under maintenance immunotherapy [13]. Current ESMO guidelines advise a stratification according to performance status and possible contraindication to immunotherapy [9]. For patients with good performance status and no contraindications, combined chemoimmunotherapy is recommended, albeit without consolidation TR. TR is considered in patients with good to moderate performance status and contraindications to immunotherapy who show a response to conventional platin-based chemotherapy. NCCN guidelines are currently the only guidelines commenting on TR as a consolidation therapy after combined chemoimmunotherapy in patients with a favorable performance status [5]. In case of complete or partial response, consideration of TR is recommended, especially in patients with residual mediastinal tumors and low extra-thoracic metastatic burden.

However, a survival benefit of consolidative TR after immunotherapy has been proposed [14, 15] and seems worthwhile pursuing, as it would combine two treatments which have been shown to improve survival in this aggressive disease. Furthermore, the combination of radiotherapy and immunotherapy has proven both safe and beneficial regarding survival benefit in non-small cell lung cancer [16–19]. Two currently ongoing prospective trials will assess the benefit of consolidative TR in patients with ES-SCLC: the TREASURE trial will assess the benefit of consolidative TR in patients without cerebral metastases [11], whereas the RAPTOR trial is employing a broader study design, testing the addition of consolidation radiotherapy—standard RT, thoracic or liver RT, and extra-thoracic RT to selective versus to all visible tumor sites—to maintenance atezolizumab, both in patients with partial response and stable disease (NCT04402788). However, the end of the recruiting phases will be 2024 and 2027, respectively, so that at present, the benefit of consolidative TR in addition to chemoimmunotherapy needs to be assessed retrospectively. Therefore, we analyzed the cohort of all patients who have received chemoimmunotherapy for ES-SCLC at our institution since the approval of PD-L1 inhibitors as first-line treatment in addition to conventional palliative chemotherapy in patients with ES-SCLC in Germany in 2019.

In our retrospective analysis, we found a significant OS benefit for patients who received TR. In patients who received TR in case of stable disease or local response, 1‑year OS was 78.6% with a median survival of 21.1 months. When adjusting for radiotherapy indication, consolidative radiotherapy was associated with a survival at 1 year of 88.9% (log-rank *p* = 0.010) and a median survival of 2.3 years (95% CI 1.25–3.35 years), compared to the CREST study, which reported a 1-year OS of 33% and a 2-year OS of 13%, corroborating a beneficial effect of consolidation radiotherapy after chemoimmunotherapy in ES-SCLC. In comparison to the chemoimmunotherapy approval studies—IMpower133, which found a 1-year OS of 51.7% with a median survival of 12.3 months [8] and CASPIAN with a median survival of 13.0 months [7]—where consolidative TR was not permitted, the addition of TR to chemoimmunotherapy provided a further survival benefit in our patient collective.

However, as our study relied on retrospective data, patients represented a heterogenous collective. Our study also comprised patients in very advanced stages of disease who died before completion of chemoimmunotherapy from cancer-related causes and were thus not considered for consolidating TR. Therefore, 12 patients were not eligible for survival analysis. In contrast to the two studies found in the literature analyzing the effect of additional TR in ES-SCLC—one retrospective study on consolidative TR [10] and one retrospective study on real-life outcomes in patients with atezolizumab, which included 4 patients with additional TR [20]—our patient collective also comprised patients who received TR in case of disease progression after initial response of the mediastinal tumor manifestation. In these patients, tumor progression occurred despite chemoimmunotherapy or maintenance immunotherapy. Here, overall survival at 1 year was 66.7% with a median survival of 1.00 year. There was no significant difference to patients receiving chemoimmunotherapy only in our study, although patients receiving TR instead of only second-line chemotherapy showed a longer median survival (12.0 months vs. 10.8 months). This OS was shorter than the OS of patients receiving TR as consolidation in case of stable disease or response to systemic treatment (median survival 27.6 months), but still compared favorably to the OS reported in the literature for patients receiving only TR [3, 4, 21] or chemoimmunotherapy alone, pointing towards a survival benefit for TR even in case of progression.

Due to the retrospective nature of our analysis and the paucity of data regarding TR after chemoimmunotherapy, the decision for or against TR was not based on a guideline which was adhered to coherently, but rather on the tumor board recommendation, patients’ wishes, and physicians’ discretion. Thus, there might be an inherent bias in the selection of patients referred to TR with regard to better performance status or tumor situation—i.e., good response to systemic therapy in general, low metastatic burden, or only local and not systemic progression—and therefore a bias in the radiotherapy indication, possibly affecting the results of our survival analysis. However, patients receiving TR as consolidation generally showed a good response of the primary tumor while TR at progression was conducted in case of progression of the primary tumor. In both groups, metastatic burden at the point of TR was either stable or, in some cases with limited metastases, also treated locally (e.g., WB-RT). A survival benefit of TR could be observed in both groups, pointing towards a benefit even in cases refractory to systemic therapy. Furthermore, TR was not routinely performed in all patients showing a good response to chemoimmunotherapy in the first re-stating (i.e., response of the primary tumor, controlled metastases), resulting in no significant difference regarding confounding factors between groups (Table 2).

As ECOG status was not routinely reported, analysis of the confounding effect of poor performance status was hindered. To adjust for this, the presence of hepatic and cerebral metastasis and, where detailed, level of NSE at primary diagnosis was recorded. In Cox regression analyses, neither the presence of cerebral nor hepatic metastases had a significant impact on overall survival while the significant association of mediastinal radiotherapy with longer overall survival was sustained. Furthermore, our study also included patients who received additional WB-RT in addition to TR. To accommodate for this imbalance, we conducted a Cox regression analysis which revealed a higher overall survival rate for patients treated with WB-RT, although this did not reach statistical significance, most likely due to the small size of the cohort (WB-RT HR 0.43, 95% CI 0.17–1.08, *p* = 0.073) and a sustained non-significant benefit for thoracic TR (HR 0.39, 95% CI 0.14–1.052, *p* = 0.063).

Data from Slotman et al. in a secondary analysis of the CREST trial suggest that hepatic metastasis also is an important factor for stratifying patients who might benefit from TR [4]. In that study, patients without hepatic metastasis had significantly longer PFS and OS. The authors reported that patients without hepatic metastasis had a significant benefit regarding OS when receiving TR, but this benefit was not sustained for patients with hepatic metastasis. In our analysis, patients without hepatic metastasis likewise had a longer OS than those with hepatic metastasis, yet this was not significant (HR 2.40; 95% CI 0.94–6.16, *p* = 0.069). However, in contrast to the data of the CREST trial, the association of TR with a longer overall survival was sustained both in the group with and without hepatic metastasis (HR 0.33, 95% CI 0.12–0.88, *p* = 0.026).

Minor toxicity due to radiotherapy treatment was not well documented, posing a limitation to our analysis. Only toxicity to atezolizumab could be assessed. Here, no difference in toxicity between groups could be found. Moreover, no treatment with immunotherapy needed to be discontinued due to pneumonitis, either with or without TR. In the only other retrospective study of TR after chemoimmunotherapy by Diamond et al. [10], no increase in radiotherapy-induced toxicity was reported, but further study is needed to assess the safety profile of additional TR in this setting.

Taken together, there are several limitations to our study due to the retrospective nature of our analysis comprising a heterogenous patient collective. As most current guidelines do not comment on TR in addition to chemoimmunotherapy, TR was not generally recommended and the decision for and timepoint of TR was not consistent across all patients. As a result, some patients received TR in case of mediastinal progression and groups of patients were not controlled for hepatic and cerebral metastasis. Cox regression analyses were therefore conducted to adjust for this possible bias. Furthermore, minor toxicities were not consistently documented.

To our knowledge, our study is the first analysis directly comparing OS in patients receiving TR in ES-SCLC with patients receiving standard-of-care chemoimmunotherapy. We found a significant OS benefit for patients who received consolidative TR. In patients receiving TR in case of progression, there was a non-significant association with longer OS. However, the conclusions drawn from the analysis of our data need to be interpreted with caution due to the small sample size, limitations, and retrospective nature of our analysis. Hopefully a survival benefit of consolidative TR in ES-SCLC will be confirmed by the recruiting prospective and randomized TREASURE and RAPTOR trials, providing the basis for offering consolidative TR as an additional effective treatment to patients with this aggressive disease.

## Conclusion

Our findings point towards a survival benefit for thoracic radiotherapy in patients with ES-SCLC with stable disease or initial response to chemoimmunotherapy. This survival benefit was more pronounced when radiotherapy was conducted after an initial response to chemoimmunotherapy, but was also sustained for patients who received radiotherapy in case of local progression. Although these findings need to be interpreted with caution due to the heterogenous patient cohort and retrospective nature of the analysis, in the absence of prospective data regarding radiotherapy indication, dose, and sequencing, our study provides a basis for offering consolidative thoracic radiotherapy in the era of chemoimmunotherapy.
